# Case Report: Dural Dissection With Ventral Spinal Fluid-Filled Collection in Superficial Siderosis: Insights Into the Pathology From Anterior-Approached Surgical Cases

**DOI:** 10.3389/fneur.2022.919280

**Published:** 2022-07-13

**Authors:** Toshitaka Yoshii, Takashi Hirai, Satoru Egawa, Motonori Hashimoto, Yu Matsukura, Hiroyuki Inose, Nobuo Sanjo, Takanori Yokota, Atsushi Okawa

**Affiliations:** ^1^Department of Orthopaedic and Spinal Surgery, Graduate School, Tokyo Medical and Dental University, Tokyo, Japan; ^2^Department of Neurology, Tokyo Medical and Dental University, Tokyo, Japan

**Keywords:** superficial siderosis, fluid-filled collection, anterior approach, dural closure, dural dissection, inner layer dural dissection in superficial siderosis 2

## Abstract

Superficial siderosis (SS) of the central nervous system is a rare disease caused by chronic and repeated hemorrhages in the subarachnoid space. Recently, attention has been paid on the association of SS and dural defect with ventral fluid-filled collection in the spinal canal (VFCC). The pathophysiology of hemosiderin deposition in patients with SS and dural defects is still unclear. However, previous studies have suggested the possible mechanism: cerebrospinal fluid (CSF) leaks into the epidural space through the ventral dural defect, and repetitive bleeding occurs from the epidural vessels that circulate back to the subarachnoid space through the dural defect, leading to hemosiderin deposition on the surface of the brain, the central nerves, and the spinal cord. Previously, the surgical closure of dural defect *via* the posterior approach has been reported to be effective in arresting the continued subarachnoid bleeding and disease progression. Herein, we describe SS cases whose dural defects were repaired *via* the anterior approach. From the direct anterior approach to the ventral dural defect findings, we confirmed that the outer fibrous dura layer is intact, and the defect is localized in the inner thin layer. From the findings of this study, our proposed theory is that dural tear at the inner dural layer causes “dural dissection,” which is likely to occur between the outer fibrous layer and inner dural border cellular layer. Bleeding from the vessels between the inner and outer Line 39–40 dural layers seems to be the pathology of SS with dural defect.

## Introduction

Superficial siderosis (SS) of the central nervous system (CNS) is a rare disease caused by chronic and repeated hemorrhages in the subarachnoid space. The subsequent deposition of hemosiderin on the brain and spinal cord surfaces leads to the development of neurological disturbance (1, 2). Progressive cerebellar ataxia, sensorineural deafness, and dementia are clinical features of SS (2, 3). The causes of bleeding include prior intradural surgery, carcinoma, vascular malformation, nerve root avulsion, and dural abnormality (2–4).

Recently, attention has been paid on the association of SS and dural defect with ventral fluid-filled collection in the spinal canal (VFCC) (5–10). The pathophysiology of hemosiderin deposition in patients with SS and dural defects is still unclear. However, previous studies have suggested the possible mechanism: cerebrospinal fluid (CSF) leaks into the epidural space through the ventral dural defect, and repetitive bleeding occurs from the epidural vessels that infiltrate into the subarachnoid space through the dural defect, leading to hemosiderin deposition on the surface of the brain, central nerves, and the spinal cord (8, 11, 12). Therefore, dural closure is considered to stop the bleeding that enters to the subarachnoid space through the defect.

Previous pieces of literature have described the surgical closure of dural defect *via* the posterior approach is effective in arresting the continued subarachnoid bleeding and disease progression (5, 6, 11–14). Herein, we describe two SS cases whose dural defects were located at the C7-T1 level and were repaired *via* the anterior approach. This is the first report of cases implementing anterior-approached dural closures. We further describe a SS case who received dural closure *via* the traditional posterior approach. From these cases, we obtained important anatomical and histological findings: The outer fibrous dura layer is intact, and the defect is localized in the inner thin layer. The SS pathology from the findings of these cases is further discussed in this study.

## Anterior-Approached Dural Closure

Written informed consent was obtained from the patients.

**Figure 1:**
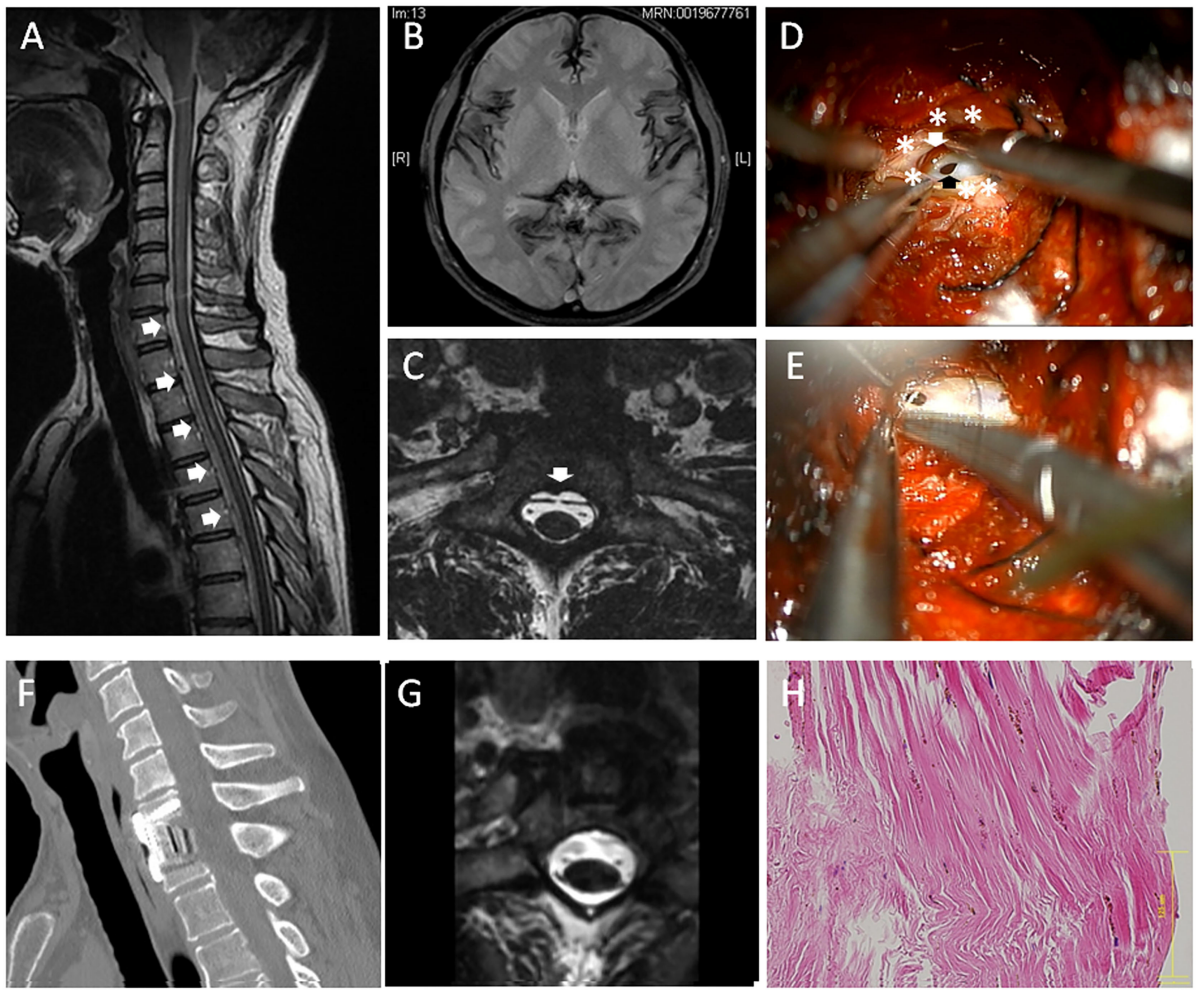
A 51-year-old male (case 1). **(A)** Sagittal magnetic resonance imaging (MRI) showed ventral fluid-filled collection in the spinal canal (VFCC) (white arrows) from C5 to T7. **(B)** Brain MRI showed a T2-weighted hypointensity in the superficial brain. **(C)** A fast imaging employing steady-state acquisition (FIESTA) image demonstrated a dural defect at the C7-T1 level (the white arrow). **(D)** An intraoperative picture of anterior approach (case 1): After the outer dura layer (white asterisks) was cut longitudinally, we found a dural defect (the black arrow) in the dura mater's inner layer. A bleeding clot was recognized between the inner and outer dural layers (the white arrow). **(E)** The dural defect was sutured using a 7-0 nylon. **(F)** The spine was reconstructed using a cage and a plate. **(G)** Post-operative MRI showed the dural defect was successfully repaired. **(H)** The outer layer of the dura was histologically examined using hematoxylin-eosin (HE) staining, showing rich collagen fibers with hemosiderin deposition. The bar: 125 μm.

Case 1: A 51-year-old male (Case 1) presented with a 5-year history of hearing loss, ataxia, unsteady gait, diplopia, and slurred speech. The patient also had dull headache. The symptoms' onset was gradual, and the clinical course was slowly progressive. The patient showed hyperactive tendon reflexes. The CSF examination showed an increased red blood cell (RBC) count (>1,000) and low pressure (10-mm H_2_O). Magnetic resonance imaging (MRI) showed a T2-weighted hypointensity in the superficial brain and along the spinal cord due to hemosiderin deposition (Figures 1A,B). Sagittal MRI image showed VFCC from C5 to T7 in the spinal canal (Figure 1B). A fast imaging employing steady-state acquisition (FIESTA) image demonstrated that a dural defect was suspected at the C7-T1 level (Figure 1C) and was confirmed by dynamic computed tomography (CT) myelography. No other abnormalities causing SS were found.

A C7 corpectomy was performed through standard left-anterior approach, and then the posterior longitudinal ligament (PLL) was resected. The T1's posterior vertebral edge was further undercut to make an appropriate space for the dural suture. The epidural space was completely exposed with approximately a 20-mm width, which revealed that the surface of outer dura mater is completely intact. Then, the dura (outer dura) was cut with a 20-mm longitudinal length, which revealed a 5-mm-long defect in the dura mater's inner layer (Figure 1D). A bleeding clot was recognized at the inter-layer between the inner and outer dural layers. The inner dural defect was closed using a 7-0 nylon suture (Figure 1E) and sealed with a fibrin glue. The inter-layer cavity was filled with mixture of muscle fragments and a fibrin glue, and then the outer layer was sutured using a 5-0 nylon. The superficial layer of the dura was histologically examined using hematoxylin-eosin staining, showing rich collagen fibers with hemosiderin deposition (Figure 1F). The spine was fused using a cage and a plate (Figure 1G). There were no perioperative adverse events. Post-operative MRI showed the dural defect was repaired (Figure 1H). Post-operatively, his headache was improved. The patient's neurological symptoms were stabilized, although a drastic improvement in the clinical manifestation was not observed. The patient was satisfied with the surgical treatment.

**Figure 2:**
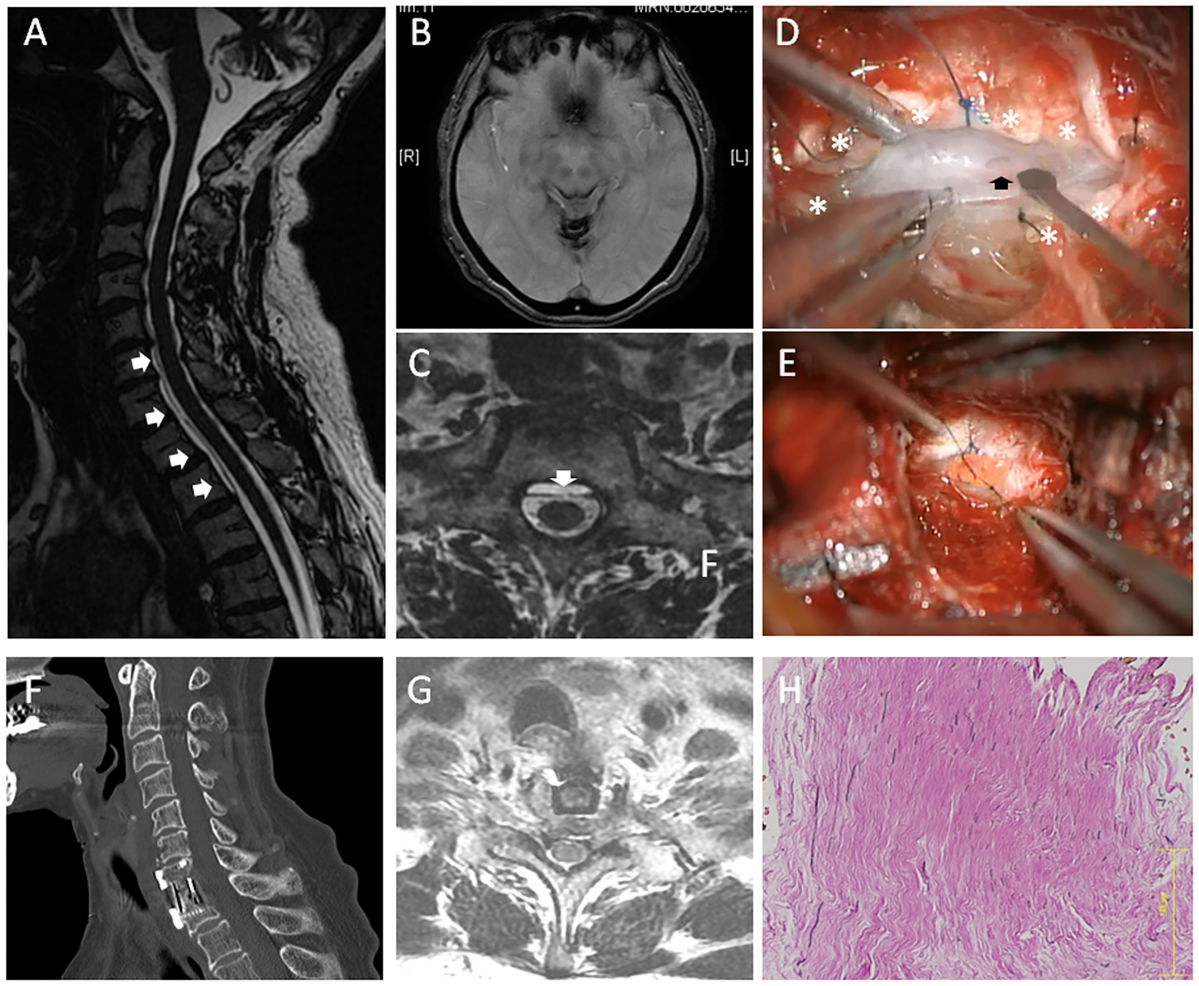
A 69-year-old female (case 2). **(A)** Sagittal MRI showed VFCC from C3 to T2 in the spinal canal (white arrows). **(B)** Brain MRI showed a hemosiderin deposition in the superficial brain. **(C)** An axial FIESTA image demonstrated that a dural defect was located at the C7-T1 level (the white arrow). **(D)** An intraoperative picture of anterior approach (case 2): After the outer dura layer (white asterisks) was excised at the center, we found a dural defect (the black arrow) in the dura mater's inner layer. **(E)** Since the inner dural layer was extremely thin and fragile, the defect was repaired using a free fat graft. **(F)** The spine was fused using a cage and a plate. **(G)** Post-operative MRI showed the dural defect was repaired. **(H)** The histology of the outer layer (HE staining) demonstrated that rich collagen fibers were oriented in a longitudinal direction. The bar: 125 μm.

Case 2: A 69-year-old female presented with a 16-year history of hearing loss, progressive gait difficulties, diplopia, and dysarthria. The symptoms were gradually progressive. The patient had a history of severe headache at the age of 27 years old. The headache continued for more than 1 year but was spontaneously resolved. The CSF examination showed an increased RBC count (>1,000). A T2-weighted MRI revealed hypointensity in the superficial brain and along the spinal cord, suggestive of hemosiderin deposition (Figures 2A,B). VFCC was observed from C3 to T2 on the sagittal images (Figure 2B). A dural defect was detected at the C7-T1 level on the axial FIESTA image (Figure 2C) and dynamic CT myelography. The imaging studies did not show any other findings causing SS.

After C7 corpectomy and partial corpectomy of T1 were performed, the PLL was resected. Similarly, the outer layer of dura was completely intact. Then, the outer dura layer's center was cut longitudinally, which revealed a 4-mm-long defect in the dura mater's inner layer (Figure 2D). Since the inner dural layer was extremely thin and fragile, the defect was repaired using a free fat graft (Figure 2E). The fat graft was placed at the defect and was sutured with the surrounding inner dura mater. Then, a fibrin glue was used for sealing. The inter-layer cavity was filled with mixture of muscle fragments and a fibrin glue, and then the outer layer was sutured using a 5-0 nylon. The histology of the superficial layer demonstrated that rich collagen fibers were oriented in a longitudinal direction (Figure 2F). The spine was fused using a cage and a plate (Figure 2G), and post-operative MRI showed the dural defect was repaired (Figure 2H). Post-operatively, the patient's neurological symptoms did not deteriorate.

## Posterior-Approached Dural Closure

Written informed consent was obtained from the patient. A 79-year-old male (Case 3) presented with a 24-year history of hearing loss, gait difficulties, and unilateral motor palsy in the left upper limb. The symptoms' onset was gradually progressive. The patient had not experienced obvious symptoms related to CSF hypovolemia. The CSF examination showed an increased RBC count (>1,000).

**Figure 3:**
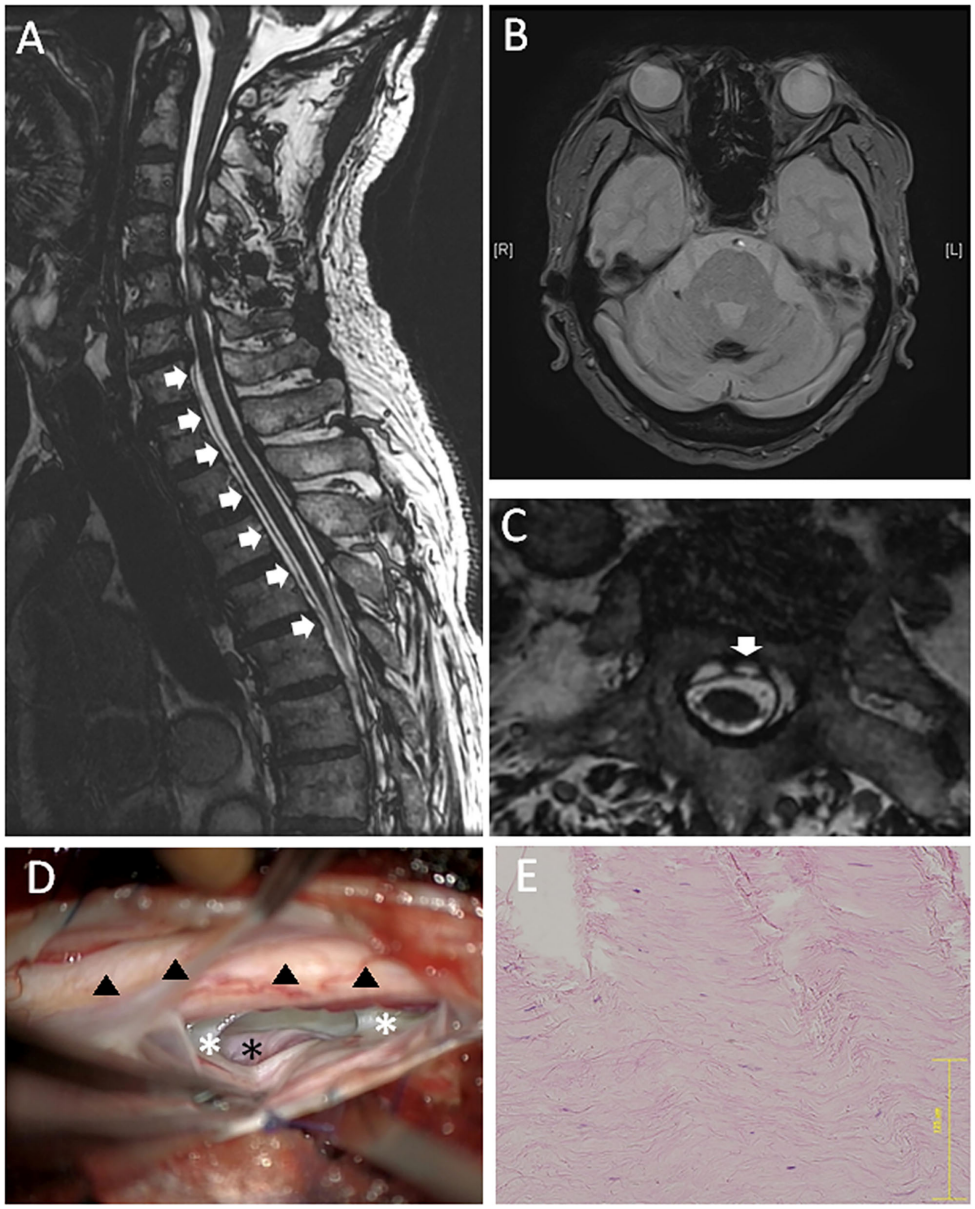
A 79-year-old male (case 3). **(A)** Sagittal MRI showed VFCC from C7 to T7 in the spinal canal (white arrows). **(B)** Brain MRI showed a hemosiderin deposition at the sulcus. **(C)** An axial FIESTA image at the T2 level demonstrated a defect located at the left paramedian anterior dura (the white arrow). **(D)** An intraoperative picture of posterior approach: The left ventral epidural space was dissected, and the surface of dura (outer dura: white asterisks) was pushed up from the anterolateral direction. The intact outer dura was observed through the dural defect, which was supposed to locate in the inner dura mater (the black asterisk). Hemosiderin deposition was observed on the spinal cord (black triangles). **(E)** The histology of the outer layer (HE staining) demonstrated rich collagen fibers. The bar: 125 μm.

A T2-weighted MRI indicated hemosiderin deposition on the superficial brain and the spinal cord (Figures 3A,B). VFCC was observed from C7 to T7 on the sagittal images (Figure 3B). A dural defect was detected at the T2 level on the axial FIESTA image (Figure 3C). There were no other findings, which could cause SS.

A posterior laminectomy was performed from T1 to T2. The dura mater was incised at the left posterolateral, site and the spinal cord was gently retracted after the dentate ligament was resected. A 6-mm vertical dural defect was identified anteriorly on the paramedian left side at T1-2 level. We dissected the ventral epidural space and pushed the surface of dura (outer dura) from the anterolateral direction (Figure 3D). Then, we observed the intact outer dura through the dural defect, which was supposed to locate in the inner dura mater. We put mixture of muscle fragments and a fibrin glue into the inter-layer cavity through the dural defect, and sutured the ventral dural defect using a 7-0 nylon. Then, the posterolateral dura was closed and sealed with a glue. Similar to the anterior-approached cases, the histology of the superficial layer demonstrated rich collagen fibers oriented in a longitudinal direction (Figure 3E). Post-operatively, neurological symptoms did not deteriorate.

## Discussions

SS of the CNS is a rare condition. The clinical features include progressive cerebellar ataxia, dysarthria, sensorineural hearing loss, bladder disturbance, and myelopathy (1–3, 15). The pathology of SS is chronic and repetitive subarachnoid hemorrhaging and a resulting hemosiderin deposition around the brainstem, the cerebellum, and the spinal cord. Recurrent subarachnoid hemorrhaging leads to the overproduction of hemoglobin-degradation products (16): toxic and unbound ferric ions accumulate when the protective mechanisms are exhausted because of chronic and repetitive hemorrhaging. It is reported that the neuronal injury was caused by subsequent free radical damage, lipid peroxidation, and membrane dysfunction (16).

Recently, there have been increasing reports of SS cases accompanied with a ventral dural defect in the spinal canal (5–10). Several authors have reported on the effectiveness of surgical defect closure for this type of SS. The pathophysiology of hemosiderin deposition in patients with SS and dural defects is still unclear. However, many authors have suggested that CSF leaks into the “epidural” space through the ventral dural defect, and repetitive bleeding occurs from the “epidural” vessels that infiltrate back to the subarachnoid space through the dural defect, leading to hemosiderin deposition in the brain, the central nerves, and the spinal cord (8, 10–12, 17). Therefore, dural closure can effectively stop not only the CSF leak but also the repetitive bleeding that enters to the subarachnoid space through the defect. Indeed, our study and others have reported that a surgical dural closure resulted in stopping of the CSF leak as well as bleeding and subsequent disease progression (5, 6, 8, 9, 14).

Surgical repair is generally performed *via* the posterior approach. After adequate levels of laminectomies are performed, the posterior dura is incised posteriorly, and the anterior dura is sutured while the spinal cord is gently retracted. When the defect is located at the anterior dura mater's center, this procedure is sometimes very difficult. Previous literature has shown that the defect is sometimes impossible to suture directly, and muscle fragments packing is performed instead (5, 14). Also, in cases with spinal cord herniation, surgeons sometimes prefer to expand the defect instead of a direct suture because of difficulty and risk of spinal cord injury (18). On the other hand, dural repair through an anterior approach can avoid spinal cord retraction, which can minimize the risk of neurological deterioration caused by surgery. If the dural defect is located at the C7-T1 level or above, surgeons can anatomically access the defect through an anterior approach and can easily make a direct suture without touching the spinal cord. Furthermore, we cannot sacrifice the C8 or T1 nerve root for spinal cord retraction. Therefore, anterior approach is more suitable for the C7-T1 level or above for safe surgery, especially in cases whose dural defect was located at the dura center. On the other hand, it is hard to anteriorly access the T2 level or below because the sternal bone anatomically disturbs the approach. A sternal splitting approach can make it possible but is associated with greater invasiveness. Therefore, if the defect is located at the T2 level or below, posterior approach is more preferable. Indeed, we selected anterior approach for Cases 1 and 2 (defects at C7-T1), but posterior approach for Case 3 (defect at T2).

We note that important findings were obtained from the anterior approach cases where we could observe the environment of the dural defect and CSF leak directly after removal of vertebrae and PLL. We observed that the dural defect was located at the dura's thin inner layer, while the thick outer layer was completely intact. As stated above, many authors have described that the bleeding occurs in the “epidural” space and infiltrates to the subarachnoid space *via* the small dural defect (8, 10, 11, 17, 19). Our findings refute these previous studies, reporting that the chronic repetitive bleeding occurs from the “epidural” vessels. Although a previous report has suggested the possibility of a “duplicate dura” in the cases of SS with VFCC (20) based on MRI images' findings, to our knowledge, no studies have proved the theory based on surgical and histological findings from anterior approach.

The dura's outermost portion was very thin membrane with less extracellular collagen, which is usually difficult to recognize during surgery. Except this thin membrane, there are two main layers of dura mater. The outer layer is the thickest portion, which is richly made up of extracellular collagen. This thick layer is called the “fibrous dura,” which we usually recognized as the dura's superficial layer (outer dura). In the inner side of the fibrous dura, there is a thin layer called the dural border cellular layer (the DBC layer), characterized by relatively few cell junctions, no extracellular collagen, and multiple enlarged extracellular spaces (21, 22). This has been suggested as the structurally weakest plane in the dura-arachnoid continuum. In our study's surgical findings, there existed a thick intact layer on the dura's outer side, and a defect was clearly detected in the inner thin layer in all these cases. The outer layer was histologically compatible to be a “fibrous dural layer” composed of rich collagen fibers. Thus, the thin inner layer is considered to be the weak DBC layer, which lacks cell junctions and extracellular collagen, where the dural defect was found. Since these DBC layers are composed of less vascularization, the defect may be difficult to repair spontaneously. Furthermore, the “epidural blood patch,” which is a CSF leak treatment method by blood injection to the epidural space, is considered to have no effect for this dural defect, because the “epidural” injection does not reach the defect located in the inner dural layer.

**Figure 4:**
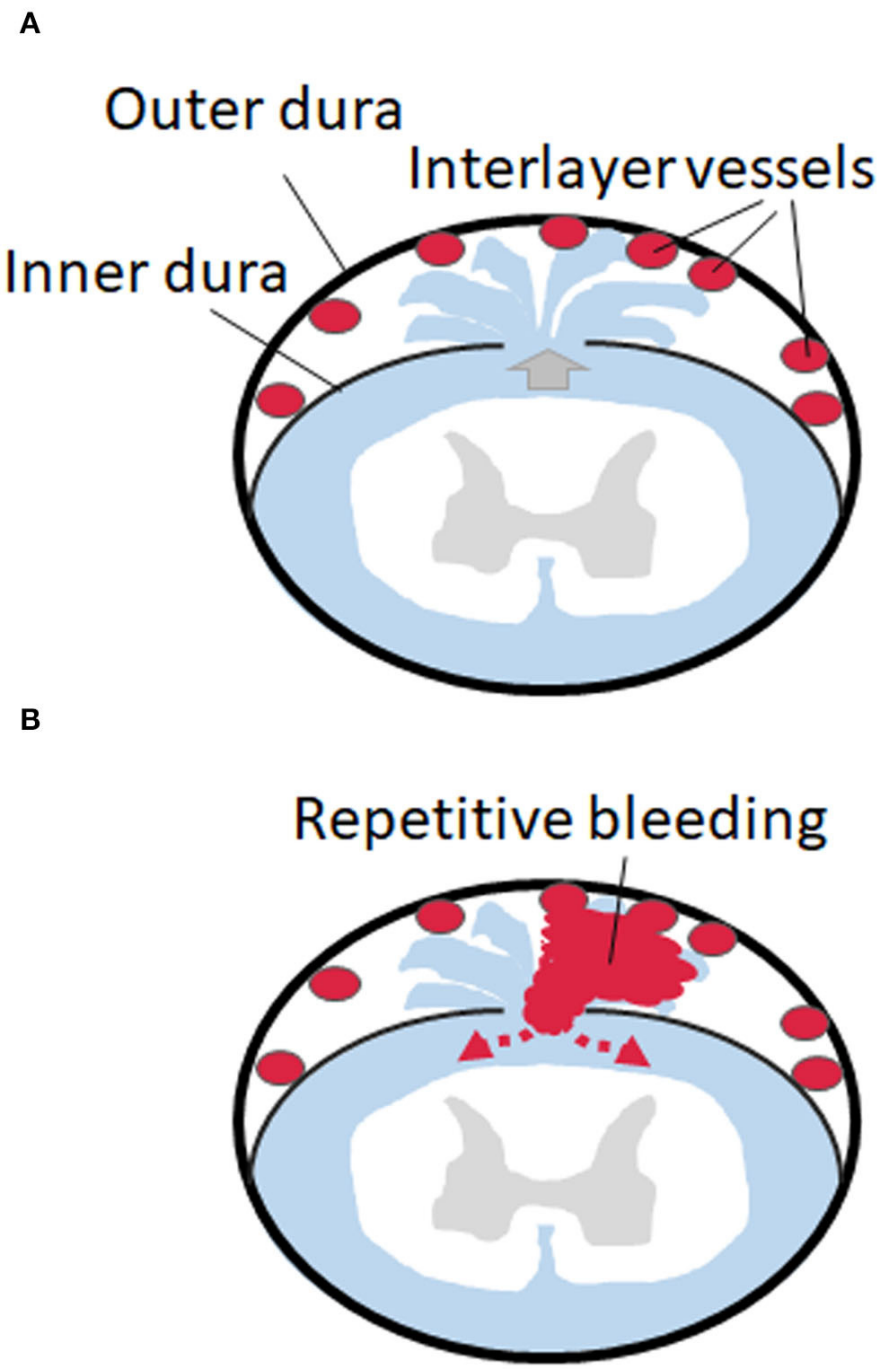
Schemes of the dural dissection and bleeding form the dissected space. **(A)** Dural tear at the inner dural layer causes “dural dissection” between the outer fibrous thick layer and the inner thin layer. A CSF leak causes venous dilatation in the inter-layer space. **(B)** The receptive dynamic CSF flow into the dissected space causes the chronic bleeding from the rich-dilated vessels localized between the inner and outer layers, which circulate back to the subarachnoid space.

Interestingly, it is known that rich vascular tissues exist in and around the fibrous dura layer (21, 22). Therefore, the pathology of an SS with spinal VFCC could be supposed as bleeding from the vessels located in the inter-dural layers (between the fibrous layer and the DBC layer) but not from “epidural” vessels. It is also known that a CSF leak can cause venous dilatation outside the arachnoid space. From the findings of this study, our proposed theory is that dural tear at the inner dural layer causes “dural dissection,” which is likely to occur between the outer fibrous layer and the inner DBC layer. The receptive dynamic CSF flow into the dissected space causes the chronic bleeding from the rich-dilated vessels localized between the inner and outer layers (Figures 4A,B). Dynamic continuous CSF flow may disturb arrest of bleeding by removing clots over the vessels. Therefore, from the cases presented in this report, dural dissection and bleeding from the inter-layer vessels, rather than the bleeding from the epidural venous plexus, are considered the true pathology of the SS accompanied with VFCC.

## Conclusion

From the direct approach to the ventral dural defect, we confirmed that the outer fibrous dura layer is intact, and the defect is localized in the inner thin layers. This finding suggests that dural dissection and bleeding from the space between the outer and inner dural layers seem to be the true pathology of SS with dural defect.

## Data Availability Statement

The raw data supporting the conclusions of this article will be made available by the authors, without undue reservation.

## Ethics Statement

The studies involving human participants were reviewed and approved by the Tokyo Medical and Dental University Ethical Committee. The patients/participants provided their written informed consent to participate in this study. Written informed consent was obtained from the patients for the publication of any potentially identifiable images or data included in this article.

## Author Contributions

TYos, TH, SE, MH, YM, and HI designed the study, collected data, analyzed the data, interpreted the data for the work, drafted the work, critically revised it, and finally approved it. NS, TYok, and AO analyzed the results, drafted and critically revised the manuscript, and finally approved it. All authors agreed to be accountable for all the aspects of the work in ensuring that questions related to the accuracy or integrity of any part of the work are appropriately investigated and resolved.

## Conflict of Interest

The authors declare that the research was conducted in the absence of any commercial or financial relationships that could be construed as a potential conflict of interest.

## Publisher's Note

All claims expressed in this article are solely those of the authors and do not necessarily represent those of their affiliated organizations, or those of the publisher, the editors and the reviewers. Any product that may be evaluated in this article, or claim that may be made by its manufacturer, is not guaranteed or endorsed by the publisher.
